# Reports of the Committee on Medical Societies and Colleges of the Senate of New-York, on Lunatic Asylums and the Education of Idiots

**Published:** 1846-05-01

**Authors:** 


*Reports of the Committee on Medical Societies and Colleges of the Senate of New-York, on Lunatic Asylums, and the education of idiots.*—Two reports by Dr. Backus, chairman of the above committee in the Senate, on the subjects mentioned, have been forwarded to us. We had prepared a synopsis of their contents, with such observations as were suggested, intended for this number. We must, however, do with it what we trust will not be done with the documents referred to—*it must lie upon the table.* The reports are creditable to the heart and head of the author. We hope the recommendations of the committee will be adopted. In no other department of legislative action can the Empire State exemplify its motto—*Excelsior*—so emphatically as in rendering its charitable institutions more and more extensive and perfect. Better far to err in doing too much than too little for such objects!

